# Accessing health services in India: experiences of seasonal migrants returning to Nepal

**DOI:** 10.1186/s12913-020-05846-7

**Published:** 2020-10-29

**Authors:** Pratik Adhikary, Nirmal Aryal, Raja Ram Dhungana, Radheshyam Krishna KC, Pramod R. Regmi, Kolitha Prabhash Wickramage, Patrick Duigan, Montira Inkochasan, Guna Nidhi Sharma, Bikash Devkota, Edwin van Teijlingen, Padam Simkhada

**Affiliations:** 1 Research Unit, Green Tara Nepal (GTN), Kathmandu, Nepal; 2 Faculty of Health & Social Sciences, Bournemouth University, Poole, UK; 3 Institute for Health & Sport (IHES), Victoria University, Melbourne, Australia; 4 International Organization for Migration (IOM), Kathmandu, Nepal; 5 Chitwan Medical College, Bharatpur, Nepal; 6 Datta Meghe Institute of Medical Sciences, Wardha, India; 7 IOM, Geneva, Switzerland; 8 IOM Regional Office for Asia and Pacific Region (ROAP), Bangkok, Thailand; 9 Ministry of Health and Population, Kathmandu, Nepal; 10 Manmohan Memorial Institute of Health Sciences, Kathmandu, Nepal; 11 Nobel College, Pokhara University, Kathmandu, Nepal; 12 School of Health and Human Science, The University of Huddersfield, Huddersfield, UK

**Keywords:** Migrants, Returnees, Healthcare access, Qualitative research, Nepal, South Asia

## Abstract

**Background:**

Migration to India is a common livelihood strategy for poor people in remote Western Nepal. To date, little research has explored the degree and nature of healthcare access among Nepali migrant workers in India. This study explores the experiences of returnee Nepali migrants with regard to accessing healthcare and the perspectives of stakeholders in the government, support organizations, and health providers working with migrant workers in India.

**Methods:**

Six focus group discussions (FGDs) and 12 in-depth interviews with returnee migrants were conducted by trained moderators in six districts in Western Nepal in late 2017. A further 12 stakeholders working in the health and education sector were also interviewed. With the consent of the participants, FGDs and interviews were audio-recorded. They were then transcribed and translated into English and the data were analysed thematically.

**Results:**

The interviewed returnee migrants worked in 15 of India’s 29 states, most as daily-wage labourers. Most were from among the lowest castes so called-Dalits. Most migrants had had difficulty accessing healthcare services in India. The major barriers to access were the lack of insurance, low wages, not having an Indian identification card tied to individual biometrics so called: A*adhaar* card. Other barriers were unsupportive employers, discrimination at healthcare facilities and limited information about the locations of healthcare services.

**Conclusions:**

Nepali migrants experience difficulties in accessing healthcare in India. Partnerships between the Nepali and Indian governments, migrant support organizations and relevant stakeholders such as healthcare providers, government agencies and employers should be strengthened so that this vulnerable population can access the healthcare they are entitled to.

**Supplementary Information:**

The online version contains supplementary material available at 10.1186/s12913-020-05846-7.

## Background

The number of Nepali who migrate to India to work continues to grow each year, in part due to the ease of crossing the open border between Nepal and India and the relative affordability of traveling there. Reliable information on cross-border mobility is not available as there is no proper reporting system, but estimates suggest that 1.6 million Nepali work in India as seasonal laborers [1]. A study conducted in Delhi shows that most Nepali perform a range of low skilled jobs, mainly as restaurant workers, factory workers, security staff, drivers, domestic workers, agriculture workers, porters, miners, rickshaw pullers, and Indian government civil servants [2].

A systematic review on health and health care of internal migrants in India suggests that migrants are at high risks of diabetes, hypertension, malaria and Human Immunodeficiency Virus (HIV) [3]. Three studies on internal migrants in India indicate that they are at risks of obesity, diabetes, hypertension, skin problems, back pain and chest pain [4–6]. Some studies have documented that Nepali migrants in India are vulnerable to many health problems, including infectious diseases such as HIV, tuberculosis (TB), and malaria [7, 8]. Studies conducted in industrialised countries show that migrants tend to experience poorer access to healthcare than the local population does [9, 10]. Promoting safer and secure working environments for all workers, including migrants is the priority of United Nations Sustainable Development Goals [11] and the timely use of personal health services is needed to achieve the best health outcomes. However, there is little evidence regarding the degree and nature of the healthcare access of Nepali migrants in India. The aim of this qualitative study is to explore the health-seeking behaviour and the barriers experienced by Nepali cross-border migrants in accessing health services in India from their perspective as well as from the related stakeholders.

## Methods

### Study design

This qualitative study is part of a larger research on “Health vulnerabilities of the cross-border migrants from Nepal” [12]. The larger mixed-methods study collected quantitative data from 751 respondents/returnee migrants. This paper is based on the qualitative study which included: (a) focus group discussions (FGDs) with migrant workers (*n* = 41); (b) in-depth interviews (IDIs) with migrants (*n* = 12) who declined to participate in FGDs; and (c) key informant interviews (KIIs) with relevant stakeholders (*n* = 12).

We carried out the FGD and interviews in six districts—Achham, Doti, Kailali, Kanchanpur, Banke and Surkhet—in Western Nepal between 2017 and 2018. Six FGDs, one in each district, were carried out. Six to eight male returnee migrants attended in each FGD [13]. In addition, 12 IDIs were conducted with returnee migrants who have more in-depth knowledge on the topic and who did not want to talk in a group setting. These returnee migrants for IDIs were selected from the 751 survey respondents. However, the key informants were not from 751 survey respondents. Key informants were the most educated person in their village to whom would-be migrant workers come for advice, and 12 KIIs were conducted with two participants from each district.

The FGD and interview guidelines were developed initially based on the available literature, in consultation with the research team and stakeholders working for migrants. These guidelines included issues such as the perception of working and living condition, perception of health risks, health problems, access to health care and barriers of accessing health care. The FGDs and interviews were facilitated by skilled qualitative researchers. The interview and FGDs guides are provided (see Additional File 1).

### Study participants

Participants were defined as “Nepali returnee migrant workers aged 18 years and over who had lived in India for at least six months for work”. Key informants included representatives from health facilities, local government offices, Non-Governmental Organisations (NGO), and International NGOs (INGOs) working on migrant issues, HIV and other health issues.

### Data organization and analysis

All FGDs and interviews were audio-recorded, transcribed and then translated into English and saved in electronic files. The researchers independently reviewed the transcripts and translated verbatim versions into English. Transcripts were cross-checked with the original recordings and subsequently analysed using a thematic approach, with themes being developed through reading the transcripts and rereading them to identify themes [14].

### Ethical consideration

Ethical approval for this qualitative study was sought from Nepal Health Research Council (Ref: 888) as part of the larger main study [12] and informed consent was obtained from all participants prior to the FGDs and interviews. A Nepali-medium ‘participant information sheet’ provided participants with information about the study, outlined the researchers’ commitment to participants’ privacy, and the confidentiality of the information collected.

## Results

The key socio-demographic characteristics of all the participants in this study are presented in (Tables 1 and 2). Their age ranged from 20 to 48 years for FGDs, 23–58 years for IDIs and KIIs. Most of the returnee migrants were labours or hotel workers and spent between 6 months to 20 years in India. Key informants were working as a health professional, NGO staff, school teacher and local leader.

**Table 1:** Characteristics of focus group participants (*n* = 41)

	FGD1	FGD2	FGD3	FGD4	FGD5	FGD6
Gender	Male	Male	Male	Male	Male	Male
Age	20–38	22–37	22–40	21–43	19–44	21–48
Ethnicity	Chhetri-8	Chhetri-3, Dalit-3	Chhetri-4 Dalit-3	Dalit-6	Dalit-8	Chhetri-4 Hill Dalit-2
Place of origin	Achham	Doti	Kailali	Banke	Surkhet	Kanchanpur
Job in India	Hotel-6 Driver-1 Security gaurd-1	Labour-6	Labour-6	Hotel-2 Labour-4	Hotel-2 Labour-4 Agriculture-1 Securityguard-1	Hotel-3 Watchman-3 Factory worker-1
State of India for work	Delhi-2 Gujrat-2, Mumbai-4	Maharastra-3 Punjab-1, Uttarpradesh-2	Delhi-2 Punjab-2, Mumbai-3	Delhi-2, Gadwal-1, Rupaidiha-1, Nanapaara-1	Maharastra-1 Gujrat-3 Mumbai-1 Simla-1 Uttarakhanda-1 Odisa-1	Mahrastra-2 Punjab-1 Kerala-2 Tamilnadu-1 Karnatak-1
Length of stay (yrs)	0.5–8.0	6.0–20	2.0–8.0	0.5–1.0	0.5–2.0	0.7–1.3

**Table 2:** Characteristics of in-depth interviewee (returnee migrants) and key informant participants (*n* = 24)

Characteristics	Returnee migrants (***n*** = 12)	KII participants (***n*** = 12)
Gender	12 male	10 male, 2 female
Age	Age range 23–58 years	Age range 23–58 years
Occupation	Labour, hotel workers, factory workers	Health professionals, NGO staff, local representatives, School teachers
Duration of work	6 months to 39 years	1–12 years
Work place in India*/ Work place in Nepal**	Mumbai-5*, Gujarat-1*, Delhi-3*, Himanchal-2*	Achham-2**, Doti-2**, Kailali-2**, Kanchanpur-2**, Banke-2**, Surkhet-2**

*This indicates work place in India for returnee migrants

**This indicates work place in Nepal for KII participants

Findings are presented under four themes: (1) accessibility, (2) perceptions, (3) affordability of healthcare services in India, and (4) barriers to accessing those services. Each theme is discussed below and relevant quotes are presented in support.

### Accessibility of healthcare in India

The FGD, KII and IDI participants reported having had mixed experiences of health services in India. Most generally agreed that health access depends on a combination of: where migrants live, the nature of the company they works for, the intelligence of the employer, their income level and local transportation facilities. About half of the KII participants mentioned that Nepali migrants struggle to get health services because they lack the proper certification:

*Without Aadhaar card, it is difficult to access health service. Those who go to India for the first time face a lot of troubles to access health services (KII, A representative of rural municipality, Achham).*

An Aadhaar card is an identification number provided to all people who live in India for more than 12 months, regardless of citizenship. The *Aadhaar* programme, which is the largest biometric identification system in the world [15], gives every cardholder easy access to various government benefits and services.

Another reason provided for limited access to healthcare was participants’ unfamiliarity with the locations of services and their struggle to make effective decisions:

*The key problem to migrant workers is the lack of information about available health services in India (KII, A health worker in Achham).*

Some participants did, however, speak positively about facilities in India:

*Most of the Nepali migrants visit government hospitals when they fall sick. In government hospital, health service is similar to what Indian get (FGD in Surkhet).*

In one FGD the efficiency of making an appointment in India was praised:

*The health centre in my place was good. It used to issue tickets even over the telephone (FGD in Surkhet).*

### Perceptions of healthcare in India

Participants were asked how healthcare workers responded to returnee migrant workers when they sought treatment at health facilities. The majority of respondents had a positive attitude towards health service delivery in India. Most felt that they had been treated fairly at Indian healthcare centers. However, a few FGD participants expressed a fear of maltreatment and some reported having encountered discrimination. A typical positive view is as follows:

*They say nothing bad to patients who go to receive treatment. They do as much as they can; otherwise, they refer them to other places (FGD in Doti).*

One participant was less positive about health workers in India:

*Indian health workers delay our treatment if we introduce ourselves as migrants (FGD in Doti).*

A returnee migrant worker who had had a health problem recalled the following experience:

*I suffered from typhoid and malaria while staying in India. I got fair treatment from the health workers. I had heard that Nepali migrant workers are dominated in the hospital but I found no such discrimination (IDI with returnee male migrant in Surkhet).*

One Nepali local leaders shared that Indian health workers do, in fact, treat Nepali workers fairly:

*Providing medical care is part of humanitarian work. So, Indian health workers treat Nepali migrant workers fairly (KII, A representative of rural municipality in Achham).*

### Affordability of healthcare in India

Migrants make choices about visiting the doctor and/or hospital depending on how much they can pay. Our study participants agreed that most migrant workers are poor and only attend government health services, although some visited private clinics in India. Nepali migrants may receive limited support from their employer towards the cost of healthcare. For example, a returnee migrant worker said this about the sharing of expenditure:

*If a company is well-established, it also bears part of the health cost. In my case, I pay myself, in hard times, I take a loan from my friends (IDI with a returnee male migrant in Doti)*

Similarly, another returnee added:

*Migrant workers approach to the government hospital to consult a doctor. One of the most frustrating of experiences is having to wait at the doctor’s office but workers are not allowing sufficient time for consultation. It’s difficult to afford private hospitals (IDI with a returnee male migrant in Kanchanpur).*

Since a small company is less likely than a big one to provide insurance coverage or cover the cost of healthcare during an illness, migrants employed by small companies are less likely to get health services in India. Some companies are very supportive, and paid for workers’ health insurance, thus:

*If workers claim medical expenses, the insurance company pays them. (IDI with a returnee male migrant in Kanchanpur).*

Indeed, in a few cases, FGD participants mentioned that they had received financial support from their employers for medical treatment in India. For example:

*When I fell sick, my company paid for me (FGD in Doti).*

### Barriers to accessing healthcare services in India

Barriers to accessing Indian health services included financial problems, language, discrimination and lack of knowledge about the location of health services. The comments below are typical.

*If you are get caught by small illness, employer will pay for you. You have to bear the cost yourself if the illness is serious (FGD in Banke).*

Others also mention language barriers and simply not knowing what was available locally:

*Neither we are confident to communicate in Indian language nor familiar about the location of health center. In such situation, it is difficult to take health services (FGD in Kanchanpur).*

Whilst unequal treatment of Nepali was also highlighted:

*Indians discriminate against Nepali people, doctors neglect us, Indians cut queues, and hospitals and doctors charge high fees (FGD in Surkhet).*

Other challenges to accessing healthcare services mentioned by several returnee migrants included the lack of information, overcrowding in government hospitals and not getting time off work from their employers for treatment:

*In India, many government hospitals are already overcrowded. In working place, it is difficult to migrant workers to get leave for doctor consultation (IDI returnee male migrant Achham).*

A number of KIIs highlighted that language barriers, delayed receipt of salaries and the passiveness of individual migrants also prevent migrants from seeking healthcare services. A health worker explained:

*In India, migrant workers are less aware of the availability of health services or health care system. Further, they experience a financial problem and also less confident to share their problems with doctors (KII with health worker in Surkhet).*

## Discussion

To the best of our knowledge, this is the first study to explore the healthcare-seeking behaviours of cross-border migrants from Nepal to India, including their use of healthcare services and the barriers to accessing those services that they face. We report three kinds of barriers: finance/cost; structural/political barriers and lack of knowledge. First, we found that access to healthcare is related to the kind of employer or company a person works for and the insurance coverage they are provided. In terms of finance, our findings suggest that it is less likely that a small company will provide insurance coverage and cover the costs of healthcare during an illness than a big company. Thus, migrants employed by small companies are less likely than working for larger big companies to get health services in India.

Like this study, a previously conducted exploratory study of Nepali migrants working in the Middle East and Malaysia highlighted that healthcare access depends on company size and the generosity of employers [16, 17]. Lee and colleagues [18] and Joshi et al. [19] also confirm the finding of our study: Indian, Bangladeshi and Myanmar migrants in Singapore and Nepali migrants in the Gulf countries had similar challenges to accessing health services as they lacked health insurance.

With regard to the cost of health during their illness in India, the majority of participants in both FGDs and interviews stated that they themselves paid to see a private doctor or return to Nepal for long-term treatment if the case was serious. These findings are similar to those of a study conducted among Chinese migrants in Singapore, which found that most of the interviewed migrants paid to see a private doctor or return to China for long-term treatment completely on their own [20]. The high cost of medical treatment for basic health services is also well documented in the literature [21–24].

Many participants reported that not having an Aadhaar card was one of the structural barriers to accessing healthcare services in India [21]. A previous study with Nepali migrants in India also reported a limited access to health and social care services by Nepali migrants due to their inability to prove their identity [25]. As many Nepali work in India as seasonal migrants and stay for less than 6 months, they are not eligible for one. Many migrant workers did not know that it is not mandatory to have an Aadhaar card to access healthcare services in government health facilities in India. In fact, migrant-related organizations facilitate easy healthcare access for those without Aadhaar cards.

Our analysis of the FGDs and in-depth interviews conducted in this study identified unfamiliarity with locations or health system in India as a reason for limited health access among migrants. Similarly, Karim and Diah [22] highlighted that Bangladeshi migrant workers in Malaysia had poor access to healthcare because the local health system was unfamiliar to them.

In the present study, among the reasons migrants were dissatisfied with the quality of health services in India were difficulty in communicating with medical staff, discrimination and delayed treatment. A few migrant workers in our study were not confident in Hindi or otherwise failed to understand the language used during their consultation. Other studies, too, have identified language as a barrier to accessing good-quality healthcare while working abroad [20, 22, 26–28].

Discrimination, too, has been reported elsewhere: two studies on minority populations in the United States (US) and a study on immigrants in Spain also highlights that these groups experienced discrimination within healthcare settings [26, 29, 30]. Another in-depth study conducted in Thailand found that Sub-Saharan African migrants living in Bangkok experienced a high level of dissatisfaction with the services provided by health professionals [31].

In contrast, a small minority of FGDs participants stated that they had positive health care experiences while being in India. Some studies also report that migrants received good-quality health care whilst working abroad [20, 32]. Possible reasons for this improvement include the lifestyle changes and health benefits that accompanied them which some migrant workers in the US experienced [33].

At a more conceptual level, our findings large fit the Health Care Access Believe Theory (HCAB) as proposed by Carrillo and colleagues [34]. This model suggests three categories of modifiable health care access barriers: financial, structural, and cognitive. Our findings reflect these three categories with perceived discrimination overarching both the structural and cognitive barriers. Discrimination is partly linked to the caste system in South Asia and perhaps less easily modifiable in Carrillo et al.’s terms [34].

The key limitation of this study is that it did not collect qualitative data (FGDs and interviews) from returnee female migrants. Since the study not being able to conduct in India meant returnee migrants were approached in their respective home districts, this will have resulted in self-selection bias. Also, the study was conducted in only six of Nepal’s 77 districts and only with returnee migrants, its findings might not be representative of the experiences of migrants in other districts or who are still working in India. The strengths of our study include giving the participants a chance to speak about experiences in India back home in Nepal with a native Nepali-speaking researcher. Also, we think FGDs are the best qualitative research approach to stimulate discussion [13], we did offer participants the opportunity to be interviewed individually.

## Conclusion

Nepali migrants in India face considerable challenges in accessing health services. Factors that limit their access include the limited healthcare services available in the vicinity, employers’ small size and limited finances, language barriers, the lack of an Aadhaar card, discrimination, and low and delayed pay. We suggest the Access Barrier Theory offers additional insights into our findings.

Nepali migrant-related organizations in India can play a crucial rule in disseminating information to Nepali migrant workers about accessing health care services in India. They can also facilitate dialogues both with healthcare service providers to minimize any existing barriers, including those stemming from language problems, as well as with employers (mainly in the formal sector) to ensure that workers are provided with health insurance coverage.

## Supplementary Information

**Additional file 1.**

## Data Availability

The transcripts used to support the conclusions of this article are available from the corresponding author on reasonable request.

## Abbreviations

AIDS: Acquired immunodeficiency syndrome
FGD: Focus group discussion
GTN: Green Tara Nepal
HIV: Human immunodeficiency virus
IDI: In-depth interviews
INGO: International non-governmental organizations
IOM: International organization for migration
KII: Key informant interviews
NGO: Non-governmental organization
NHRC: Nepal health research council
TB: Tuberculosis
UN: United Nations
