# Supplementary material for: Accessing health services in India: experiences of seasonal migrants returning to Nepal
Source: BMC Health Serv Res. 2020 Oct 29;20:992. doi: 10.1186/s12913-020-05846-7 (PMC7597057; doi:10.1186/s12913-020-05846-7)
Supplement: Supplementary file 1 — Additional file 1. [file 12913_2020_5846_MOESM1_ESM.doc]

**Health Vulnerabilities of the Cross Border Migrants From Nepal**

**Focus Group Discussion Guide**

**Socio Demographic characteristics:**

Name of facilitator/moderator: Date:

Gender:

| S. no. | Age | Marital Status | Highest level of Education | Ethnicity | Occupation | The place in India | Duration of work  (in month) |
| --- | --- | --- | --- | --- | --- | --- | --- |
| 1 |  |  |  |  |  |  |  |
| 2 |  |  |  |  |  |  |  |
| 3 |  |  |  |  |  |  |  |
| 4 |  |  |  |  |  |  |  |
| 5 |  |  |  |  |  |  |  |
| 6 |  |  |  |  |  |  |  |
| 7 |  |  |  |  |  |  |  |
| 8 |  |  |  |  |  |  |  |
| 9 |  |  |  |  |  |  |  |
| 0 |  |  |  |  |  |  |  |

[**Eligibility**: Participant must be 18 years old, have lived abroad for at least 6 month as a labour migrant and has agreed to take part this study]

**Ground rules to facilitate the discussion**

- Only one person speaks at a time. There may be a temptation to jump in when someone is talking but please wait until they have finished.
- There are no right or wrong answers.
- You do not have to speak in any particular order.
- When you do have something to say, please do so. There are many of you in the group and it is important that I obtain the views of each of you.
- You do not have to agree with the views of other people in the group.
- Please answer if there is any query from participants.

**Introductory question**

I am just going to give you a couple of minutes to think about your living experience while you work as a labour migrant in India. To begin, could you please share me your opinion about how was your life in Nepal before you went abroad? What attracted/motivated you to go there?

**Guideline for the discussions (These are the main questions. Facilitators might ask supporting questions or give examples to penetrate issues)**

**Please tell me the situation of migrant workers in your district/locality**

- What are the major destination countries for migrants in your area?
- Why are Nepali people seeking employment in India?

(prompt: main push/pull factors)

**What is the living condition of Nepali migrants in India?**

- Where do you (Nepali migrants) live? (Prompt: facilities at apartment/quality of apartment/number of people living at same apartment)
- How is your living condition? (Is it better than Nepal?)
- How do migrants entertain/fun?

**Please tell me the working situation of migrant workers in India?**

- What types of work are migrants (including yourself) doing?
- What about work environment?
- What are the facilities at work place?

(Prompt: break time, Snacks, lunch, tea, over time etc etc.?

- Are you discriminated, misbehaved at work?

**Can migrants (you) get health-related information in India?**

- What kinds of health information did you receive?
- Who provides this information? (Prompt: television, radio, newspaper, magazine, leaflet, poster etc.)

**What are the key health problems/risks to migrants (including yourself) in India?**

- What are the main health problems specific to Nepali migrants?
- Provide some example of health problems.

**Can migrants (you) go for the treatment during their illness?**

- What is your view of accessibility of services for migrants (including yourself)?
- How do they find out about the health services?
- Who pay for them (you)?
- Are there any health care services specially targeted to migrant workers?

**What are the key barriers of getting health services in India?**

- Provide some examples of barriers.

(Prompt: language barrier, lack of information, high cost etc etc)

**How can Nepali migrants be better protected when migrating for work?**

- Who should be responsible?
- How can migrants be better informed to reduce risk of poor experiences and outcomes?
- What kind of controls need to be implemented?

Is there anything else you would like to say about this?

Many thanks for your help!

Do you like to ask any questions or anything you like to discuss which we did not talk?

Thank you for your active participation!

**Health Vulnerabilities of the Cross Border Migrants From Nepal**

**Guideline for In-depth Interviews**

Date:

Name of interviewer/moderator:

Place of interview:

Age:

Sex:

Destination country:

**Key questions:**

**Guideline for the interview (These are the main questions. Facilitators might ask supporting questions or give examples to penetrate issues)**

**Please tell me the situation of migrant workers in your district/locality**

- What are the major destination countries for migrants in your area?
- Why are Nepali people seeking employment in India?

(prompt: main push/pull factors)

**What is the living condition of migrants (including yourself) in India?**

- Where do you live? (Prompt: facilities at apartment/quality of apartment/number of people living at same apartment)
- How is your living condition? (Is it better than Nepal?)
- How do you entertain/fun in India?

**Please tell me the working situation of migrant workers (including yourself) in India?**

- What types of work are migrants (including yourself) doing?
- What about work environment?
- What are the facilities at work place?

(Prompt: break time, Snacks, lunch, tea, over time etc etc.?

- Are you discriminated, misbehaved at work?

**Can migrants (you) get health-related information in India?**

- What kinds of health information did you receive?
- Who provides this information? (Prompt: television, radio, newspaper, magazine, leaflet, poster etc.)

**What are the key health problems/risks to migrants (including yourself) in India?**

- What are the main health problems specific to Nepali migrants?
- Do (or did) you have (or had) any health issues?
- Provide some example of health problems.

**Can migrants (you) go for the treatment during their illness?**

- What is your view of accessibility of services for migrants (including yourself)?
- How do they (you) find out about the health services?
- Who pay for them (you)?
- Are there any health care services specially targeted to migrant workers?

**What are the key barriers of getting health services in India?**

- Provide some examples of barriers.

(Prompt: language barrier, lack of information, high cost etc etc)

**How can Nepali migrants be better protected when migrating for work?**

- Who should be responsible?
- How can migrants be better informed to reduce risk of poor experiences and outcomes?
- What kind of controls need to be implemented?

Is there anything else you would like to say about this?

Many thanks for your help!

Do you like to ask any questions or anything you like to discuss which we did not talk?

Thank you for your active participation!

**Health Vulnerabilities of the Cross Border Migrants From Nepal**

**Guideline for Key Informant Interviews (KII) with Stakeholders**

***(health workers, local leaders, NGO staff, school teacher, policymakers)***

Date:

Name of interviewer:

Place of Interview:

Age:

Sex:

Stakeholder category:

Health Care Provider □ Local leaders □ Policy maker □ NGO staff □ School teacher

**Key questions**

- How do you view the situation of migrant workers in your locality?

(Prompt: where do they go?/ Key drivers of migration?/male/female pattern)

- Which countries are the major destination for the migrants of your area? And why people choose those destinations?
- In your opinion, what are the key health problems of Nepali migrants who migrate to India for work?
- Please provide any specific evidence/information regarding health problems faced by migrant workers?
- What are the key barriers for migrant workers to seek health care service in India (and Nepal)?
- Do you think health seeking behavior among migrant workers to India is different than the local counterparts? If yes, would you please explain it further?
- Do you or your organization ever have involved to tackle with health problems face by migrants? If yes, please mention the efforts you have made.
- Is there any provision in your locality to control cross border transmission of the diseases?
- Is there any cooperation among inter-border health service provider agencies to tackle with the health vulnerabilities of migrant workers?
- Are there any mechanisms in your villages to control and resolve the health problems faced by migrant workers after they return back?
- Are there any awareness activities conducted focusing the health problems faced by migrants?
- In your opinion what are the major steps to be undertaken to control cross-border transmission of disease and to prevent migrants from health vulnerabilities in both India and Nepal?
- Finally, do you have any suggestions to resolve health issues of migrant workers?
- Do you like to add any points we did not include?
